# The study on the early warning period of varicella outbreaks based on logistic differential equation model

**DOI:** 10.1017/S0950268818002868

**Published:** 2019-01-25

**Authors:** Feng-Rui Pang, Qing-Hong Luo, Xiu-Qin Hong, Bin Wu, Jun-Hua Zhou, Wen-Ting Zha, Yuan Lv

**Affiliations:** Key Laboratory of Molecular Epidemiology of Hunan Province, School of Medicine, Hunan Normal University, Changsha 410081, China

**Keywords:** Logistic equation, mathematical model, ordinary differential equation, varicella, warning

## Abstract

Chickenpox is a common acute and highly contagious disease in childhood; moreover, there is currently no targeted treatment. Carrying out an early warning on chickenpox plays an important role in taking targeted measures in advance as well as preventing the outbreak of the disease. In recent years, the infectious disease dynamic model has been widely used in the research of various infectious diseases. The logistic differential equation model can well demonstrate the epidemic characteristics of epidemic outbreaks, gives the point at which the early epidemic rate changes from slow to fast. Therefore, our study aims to use the logistic differential equation model to explore the epidemic characteristics and early-warning time of varicella. Meanwhile, the data of varicella cases were collected from first week of 2008 to 52nd week of 2017 in Changsha. Finally, our study found that the logistic model can be well fitted with varicella data, besides the model illustrated that there are two peaks of varicella at each year in Changsha City. One is the peak in summer–autumn corresponding to the 8th–38th week; the other is in winter–spring corresponding to the time from the 38th to the seventh week next year. The ‘epidemic acceleration week’ average value of summer–autumn and winter–spring are about the 16th week (ranging from the 15th to 17th week) and 45th week (ranging from the 44th to 47th week), respectively. What is more, taking warning measures during the acceleration week, the preventive effect will be delayed; thus, we recommend intervene during recommended warning weeks which are the 15th and 44th weeks instead.

## Introduction

Chickenpox, a characteristic vesicular rash, is a respiratory infection caused by the varicella-zoster virus which can be transmitted through airborne droplets and through the contact with the patient's fresh blisters or mucosal secretions [1]. Meanwhile, it is a common acute and highly contagious disease during childhood, and 90% [2] of susceptible children will be infected and most of which will not be seeking medical attention. However, serious complications mainly including neurological involvement and secondary bacterial infections and even severe death were observed both among cases of children and adults. The risk for disease increases with age but is also elevated in infants and the hypoimmunity [3, 4]. The present study focuses on the analysis of the vaccine efficacy and does not effectively prevent the occurrence and outbreak of chickenpox. Therefore, carrying out an early warning of chickenpox plays an important role in taking targeted measures in advance as well as preventing the outbreak.

Studies [5–8] have indicated that the logistic differential equation model can well demonstrate the epidemic characteristics of epidemic outbreaks, give the point where the early epidemic rate change from slow to fast, and is suitable for the simulation of epidemic features in epidemic cycles of infectious diseases. What is more, the study has illustrated that chickenpox has significant seasonality and periodicity; thus, it is suitable for pre-seasonal warning research [9]. Therefore, our study intends to use the logistic differential equation model to fit the weekly data of chickenpox in Changsha from 2008 to 2017, to discuss the application of the model, calculate the inflection point of epidemic speed changes in each period of chickenpox and get the warning week finally, and thus, provide a scientific basis for early warning of chickenpox.

## Materials and methods

### Data collection

The data used in this study were obtained from the Chinese Information System for Disease Control and Prevention. We collected all reported cases of chickenpox in Changsha from the first week of 2008 to 52nd week of 2017. The diagnostic criteria are in accordance with the Diagnosis and Testing (Herpes Zoster) for chickenpox [10, 11].

### Model of the logistic differential equation

The logistic differential equation model was originally put forward by *Verhust* [8] in 1845 which is an ordinary differential equation used to describe the self-growth characteristics of populations. The model differential equation is as follows:1
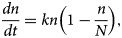
where *dn*/*dt* denotes the rate of change of cumulative cases (*n*) at time *t*, *k* is the model correlation coefficient and *N* is the cumulative case upper limit of infectious diseases. While *dn*/*dt* = *kn* is a kind of Malthusian model, and its epidemiological significance is: accumulated cases increase exponentially over time. The effect of 1 − *n*/*N* is to adjust the Malthusian model, which can vary from 0 to 1. When *n* tends to 0, 1 − *n*/*N* is close to 1, indicating that the logistic model approximates the Malthusian model. With the prevalence of the disease, due to the establishment of the population's immune barrier and the saturation of pathogens in the host population, the prevalence of the disease will tend to be stable, and the number of new cases will decrease until the epidemic tends to end. Therefore, the curve of the logistic model shows the classic ‘slow-fast-slow’ trend, which is just like the ‘*S*’ type.

Solving equation (1):2
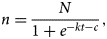
where *c* is a fixed constant.

### The point of inflection

The curve of the logistic differential equation model changes from slow to fast, and the turning point is defined as the inflection point. Applying the method of doing the third-order derivation over equation (2) to make the new equation equals to zero.3
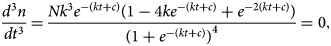
and the result is *t* = ( − *c* ± 1.317/*k*), while
4
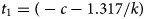
is the inflection point of the curve of the logistic equation model.

### Mathematical simulation and processing

**Fig. 1.**
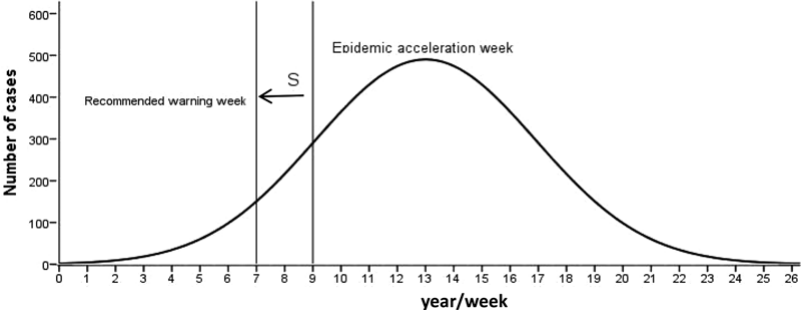
Diagram of the method to determine early warning week of varicella in epidemic cycle (S is the standard deviation).

The first step is using the Excel software to organise the data; then, the epidemic characteristics and periodicity was analysed. Logistic differential equation model of each cycle was established according to varicella epidemic characteristics; moreover, the parameter *k* and *N* were calculated by the incident cases of actual weeks. Then, the parameter *c* in equation (2) is obtained through the introductory method in mathematics, and the inflection point of the prevalence change of each epidemic period, that is, ‘epidemic acceleration week’, just like Figure 1 shows. Finally, we describe the central and dispersion trends of epidemic acceleration week in each epidemic period through corresponding statistical indicators.

### Simulation method

Berkeley Madonna 8.3.18 was employed for model simulation, and the Microsoft Excel 2007 was used for data management and figures. In addition, the fourth-order Runge–Kutta method, with tolerance set at 0.001, was used to solve the differential equation. The root mean square between the data and best run was used to judge the goodness of fit [12].

## Results

### The epidemic characteristics of chickenpox

**Fig. 2.**
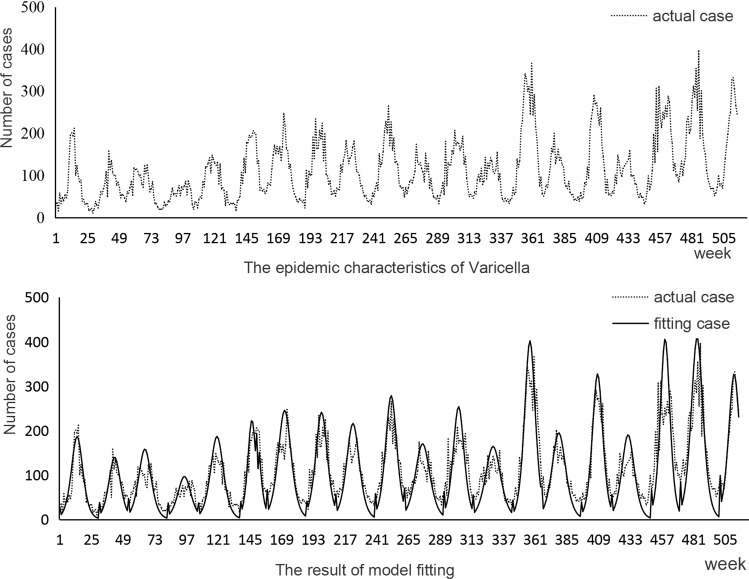
The epidemic characteristics and the result of logistic model fitting of varicella in Changsha of China from 2008 to 2017.

It can be seen from Figure 2a that there are two epidemic peaks of chickenpox every year in Changsha, including peaks in summer–autumn and winter–spring. The corresponding time is, respectively, from the eight to the 37th week of each year, and from the 38th to the seventh week of the next year. While there were 53 weeks in 2009 and 2015, the 38th week of 2009 to seventh week of 2010 include 23 weeks, and the other epidemic periods are 22 weeks.

### The fitting effect of the logistic differential equation model

The reported data were simulated by logistic differential equation. It can be seen that the simulated results agreed well with the reported (Fig. 2b), that is, the fitting effect is better. In addition, the parameters of *k*, *N* and *c* of the summer–autumn and winter–spring for each model year are shown in Table 1.

**Table 1.** The parameters of the logistic model fitting results of varicella epidemic in summer–autumn and winter–spring between 2008 and 2017 in Changsha

Time	Parameter *k*	Parameter *N*	Parameter *c*
Summer–autumn			
2008	0.346	2162	−4.68
2009	0.317	2002	−4.07
2010	0.309	2415	−4.47
2011	0.304	3235	−4.16
2012	0.311	2776	−4.18
2013	0.271	2518	−3.83
2014	0.262	2510	−4.06
2015	0.290	2697	−3.84
2016	0.318	2399	−4.05
2017	0.336	4880	−4.37
Winter–spring			
2008	0.341	1642	−4.08
2009	0.299	1291	−3.82
2010	0.423	2522	−4.55
2011	0.338	2858	−3.92
2012	0.351	3179	−4.34
2013	0.362	2804	−4.13
2014	0.370	4348	−4.98
2015	0.384	3425	−4.85
2016	0.415	3934	−4.49
2017	0.356	3679	−3.94

### Determination of the warning week

The parameters are plugged into equation (4) to calculate the epidemic acceleration week for each epidemic period. The epidemic acceleration week for different seasons in each year is shown in Figure 2b. However, the week of the accelerated epidemic is calculated by the model, and the number of cases accounted for an average of 30% (25–35%) of the cumulative number of cases in the current year. The corresponding actual epidemic had developed to a higher level, which means that if the warning measures were taken during the acceleration week, there will be a delay of the preventive effect.

**Fig. 3.**
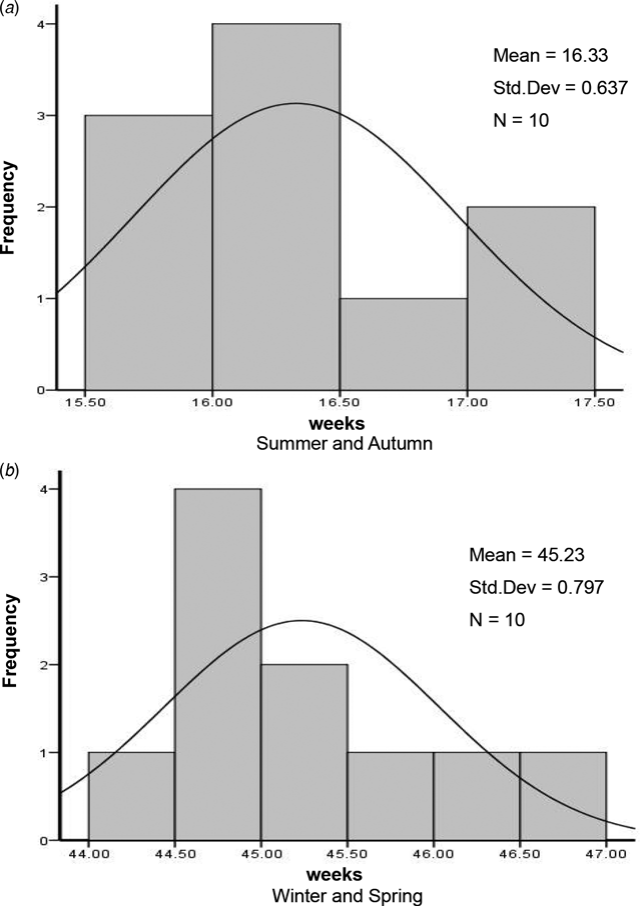
‘Epidemic acceleration week’ histogram of varicella in summer–autumn and winter–spring in Changsha.

By analysing the epidemic acceleration week in the same seasons of each year, it can be known that the average ‘acceleration week’ in summer–autumn is around the 16th week (range: 15–17th week), the standard deviation is about one week; while the average ‘acceleration week’ in winter–spring is about the 45th week (44–47th week), and the standard deviation is about one week (Fig. 3).

**Fig. 4.**
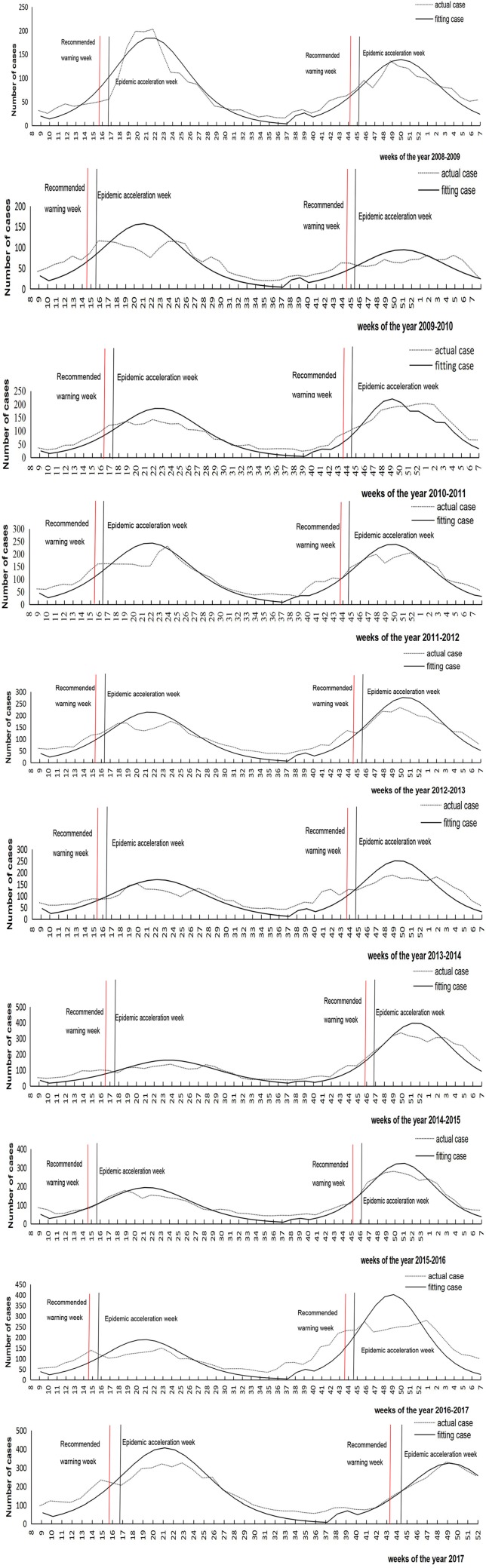
‘Epidemic acceleration week’ and ‘recommended warning week’ of varicella in summer–autumn and winter–spring in Changsha city from 2008 to 2017.

Therefore, it is recommended to take one standard deviation ahead of the acceleration week as the average warning week, that is, the 15th and the 44th weeks of each year (Fig. 4). From Fig. 4, we can get a conclusion that the proposed warning weeks from 2008 to 2017 are all in the early stages of the epidemic; thus, there is a possibility that we can provide early warning at these weeks.

## Discussion

Chickenpox is distributed globally which is a common infectious disease among children. In addition, it has a very strong ability to transmit among the population, and can affect nearly 90% of children [2]. China has begun to report varicella outbreaks through the ‘China Disease Prevention and Control Information System’ since 2005.

Although varicella-infected patients are more common in light and moderate cases, if left untreated, the infection can lead to post-herpetic neuralgia, bacterial infections and, ultimately, death [13]. At present, there is relatively less research on varicella, and the measures of prevention still stay in the stage of control and symptomatic treatment.

Many studies showed that there are many models for the prevention and prediction of infectious diseases, such as time-series models, grey models and SIS models based on complex network theory [14–18]. At the same time, studies have shown that the logistic differential equation model can also be used to fit and predict the epidemic situation of infectious disease, which can also predict the early warning period for seasonal infectious diseases.

In this paper, the epidemiological characteristics of varicella were studied by logistic differential equation model and the early warning week was obtained. The results show that chickenpox in Changsha City has obvious periodicity and seasonality, and the epidemic cycle starts from the 8th week of each year to the 7th week of the next year. Therefore, it is recommended that chickenpox surveillance and statistical methods should be adapted for their prevalence characteristics. In addition, the research results show that the logistic model can well fit the epidemic pattern of chickenpox, and we can get the ‘acceleration week’ of winter–spring and summer–autumn, respectively, each year according to the model. However, it can be seen that the epidemic has reached a high level when the epidemic has accelerated, which means that there will be a lag if the warning measures were taken during the acceleration week. Therefore, ‘recommended warning week’, calculated based on the standard deviation, is a better-recommended warning time.

In practice, it usually takes a certain amount of time to show the effect of the implementation of interventions [19]. Therefore, the varicella intervention usually needs to be planned and implemented early. This means that the early warning signal of chickenpox needs to be issued in advance to provide sufficient preparation time for the health decision-making and the measures implementation departments. The ‘advanced warning week’ proposed in this study is 2 weeks prior to the epidemic season which is the timing that the rate of change of the epidemic progresses from slow to fast. This can provide sufficient time for preparation and implementation of interventions.

In this study, the logistic differential equation model was used to analyse the prevalence and periodicity of varicella epidemics in Changsha City from the eight week of 2008 to the 52nd week of 2017; to establish the logistic differential equation model in each cycle according to the epidemic pattern of chickenpox, and further calculation of the epidemic development rate parameter *k* and the theoretical value *N* of cumulative cases at the end of the epidemic cycle through the actual weekly epidemiological data. In addition, we get the ‘epidemic acceleration week’ and ‘recommended warning week’ of chickenpox, which provided a scientific basis for better understanding of the epidemic characteristics and early warning work of chickenpox. Of note, although our study has achieved its original purpose, it still has certain limitations due to the limitations of the model and the data availability. First, the model is based on epidemiological data analysis without considering the characteristics and the climatic condition of the disease, omitting them from the model may have slightly affected the simulated data. Second, the effect of the implementation of the interventions were not added when the warning week determined. In the future, these dynamic characteristics, intervention effects and climatic factors can be added to conduct in-depth studies to provide more detailed evidence for the prevention and control of chickenpox.

## Conclusions

The varicella in Changsha has a clear cycle and seasonality, and there are two peaks each year: winter–spring and summer–autumn. What is more, the logistic model can be well fitted with varicella data. However, the early warning week obtained by the model shows that the warning has lagged. Therefore, adjusting by the standard deviation can give a better-recommended warning time, and this has a good effect on preventing chickenpox outbreaks and can provide a scientific evidence for the clinical and early warning work of Varicella.
